# Burden of *Neisseria meningitidis* infections in China: a systematic review and meta–analysis

**DOI:** 10.7189/jogh.06.020409

**Published:** 2016-12

**Authors:** Yaowen Zhang, Dong Wei, Xinzhen Guo, Mai Han, Lichao Yuan, Moe H Kyaw

**Affiliations:** 1 Infection Management and Disease Prevention Department, China–Japan Friendship Hospital, Hepingli, Beijing, China; 2 Department of Infectious Diseases, China–Japan Friendship Hospital, Hepingli, Beijing, China; 3 Sanofi Pasteur, Discovery Drive, Swiftwater, PA, USA

## Abstract

**Background:**

*Neisseria meningitidis* is a leading cause of bacterial meningitis and septicemia in children and young adults worldwide. The disease burden associated with *N. meningitidis* infections has not been systematically assessed in China. Therefore, we undertook this study to determine the burden of meningococcal disease in China.

**Method:**

We performed a systematic review and meta–analysis of articles on *N. meningitidis* incidence, carriage, seroprevalence and mortality rates in China by searching the Chinese BioMedical Database (CBM), China National Knowledge Infrastructure (CNKI), Wanfang database and PubMed for publications from January 2005 to Aug 2015.

**Results:**

In total, 50 articles were included in our analysis. The overall incidence of meningococcal disease and associated mortality were estimated to be 1.84 (95% confidence interval (CI) 0.91–3.37) per 100 000 persons per year and 0.33 (95% CI 0.12–0.86) per 100 000 persons per year, respectively. *N. meningitidis* carriage rate among the healthy population was estimated to be 2.7% (95% CI 2.0–3.5%). Prevalence of antibodies against *N. meningitidis* serogroup A and C were estimated to be 77.3% (95% CI 72.4%–81.6%) and 33.5% (95% CI 27.0%–40.8%), respectively. No studies were found for serogroup specific disease burden.

**Conclusions:**

The overall incidence of meningococcal disease in China is low. The lower seroprevalence of serogroup C within the population suggests that it may pose a greater risk for meningococcal disease outbreak than serogroup A. The lack of data on serogroup disease burden by age groups suggests the implementation of laboratory based meningococcal surveillance systems are urgently needed in China.

*Neisseria meningitidis*, a Gram–negative diplococcus, asymptomatically colonizes the upper respiratory tract of approximately 10% of healthy humans, and is a leading cause of bacterial meningitis and septicemia in children and young adults worldwide [1,2]. It is transmitted through direct contact with respiratory secretions or aerosol droplets released by coughing/sneezing from patients with meningococcal disease or asymptomatic carriers. Humans are the only host [3]. Most cases of meningococcal disease are caused by serogroups A, B, C, Y and W [4]. Meningitis caused by *N. meningitidis* continues to be a serious threat to global health, accounting for 1.2 million cases and 135 000 deaths worldwide each year [5], despite the existence of effective vaccines [6].

Serogroup A has historically been the dominant serogroup in China accounting for over 95% of meningococcal disease cases from the 1960s to 1980s, with annual disease incidence rates up to 400 cases per 100 000 population in some regions [7,8]. Following the introduction of a serogroup A polysaccharide meningococcal vaccine into the national immunization program in 1982, the incidence rates in the subsequent two decades were reduced and remained low and relatively stable ranging 0.2–1 cases per 100 000 [9,10]. However, during 2003–2004, serogroup C (type ST–4821) meningococcal disease outbreaks emerged in the Anhui province [11]. This new strain appeared more invasive, causing serious complications more frequently and was associated with a higher case–fatality rate than serogroup A [12]. Serogroup C meningococcal disease quickly became endemic in the Anhui Province, with ST–4821 the dominant lineage. This lineage rapidly spread nationwide causing several meningococcal disease outbreaks in 2004–2005 [12,13]. In response to these outbreaks, meningococcal group A and C polysaccharide vaccines were subsequently used for routine immunization. Nonetheless, serogroup C continues to be isolated every year throughout China with ST–4821 dominant [14].

Understanding changes in epidemiology of meningococcal disease after use of meningococcal A/C polysaccharide vaccines and *N. meningitidis* carriage rates and seroprevalence can help predict the potential public health impact of routine vaccination. To date, the disease burden associated with *N. meningitidis* infections has not been systematically assessed in China. We therefore conducted this systematic review and meta–analysis to evaluate the incidence of meningococcal disease and associated mortality, as well as carriage rates and prevalence of antibodies against *N. meningitidis* in China.

## METHODS

### Search strategy

We undertook a systematic search across the following electronic databases: Chinese BioMedical Database (CBM), China National Knowledge Infrastructure (CNKI), Wanfang database and PubMed. The specific details of the search strategies undertaken across these databases are presented in Appendix S1 in **Online Supplementary Document(Online Supplementary Document)**. In brief, the following search terms were used to search the databases: “meningococcus”, “meningococcal”, “meningococcic” and “*meningitidis”*. The search in PubMed included ‘China’ as a search term. We restricted our search to articles published in Chinese “core journals”, as listed by the Peking University (2014 edition) and evaluated according to predefined criteria; journals considered to be of low quality are excluded from the list [15]. We focused our search on recent data, published from January 2005 to August 2015.

### Inclusion and exclusion criteria

Studies were selected for inclusion based upon the following criteria: 1) included humans; 2) reported at least one outcome relevant to our study objectives; 3) published in Chinese or English language. We excluded case reports and other systematic reviews or meta–analyses. Where multiple studies on the same cohort were identified, the latest publication or that with the most complete data was included in our meta–analysis.

### Literature screening and data extraction

Two groups of reviewers screened the titles, keywords and abstracts of the citations identified (DW and XG independently reviewed records 1–1850; MH and LY independently reviewed records 1851–3703) and excluded those that clearly did not meet the inclusion criteria. YZ screened the citations identified in a similar manner. The full texts of all selected publications were assessed for relevance. Any disagreement or uncertainty between the reviewers about the eligibility of a study was resolved by YZ, and in the case of persistent disagreement, the full text of the article was examined. The reference lists of articles identified for inclusion were inspected for other appropriate articles not identified by the electronic search.

The reviewers independently extracted and entered data from each included study into a database. The following data were extracted from the studies where available: authors; year of publication; study design; study period; location of the study; number of meningococcal disease cases or reported incidence (crude and age–specific); number of deaths or reported death rates; *N. meningitidis* carriage rates; and prevalence of antibodies against *N. meningitidis* and corresponding serogroup. The data extracted were checked for inconsistencies between the reviewers and resolved by a fifth author. We did not attempt to contact the authors of the studies identified for missing information or resolve ambiguities.

### Quality assessment

The analysis included studies with different outcomes. Therefore, no pre–existing scale is directly suit able for the quality assessment. The quality of each included study was assessed by YZ and DW using predefined criteria as previously described [16]. In brief, the quality of the studies was based on the clarity of information provided on the following 5 items scored on a three–point scale (from 0 – poorest to 2 – best quality): population and representativeness; diagnostic criteria; specimen collection methods; pathogen or antibody detection methods; and statistical methods. The scoring is defined as 2 points for detailed reporting, 1 point for non–detailed reporting and zero point for no reporting of the selected criteria for the assessment. The score for each item was then added to give a composite score for the study, with a highest total score of 10. Studies with total scores ≥8 were regarded as “good” quality.

### Statistical analysis

All meta–analyses were performed using the MetaAnalyst (Beta 3.13; http://tuftscaes.org/meta_analyst) software package. Since we were expecting considerable heterogeneity across the included studies, we used the random–effects model of the Der–Simonian Laird method. Publication bias was investigated via Stata 12.0 (StataCorp LP, Texas, USA) using Egger’s test.

## RESULTS

### Studies included

**Figure 1:**
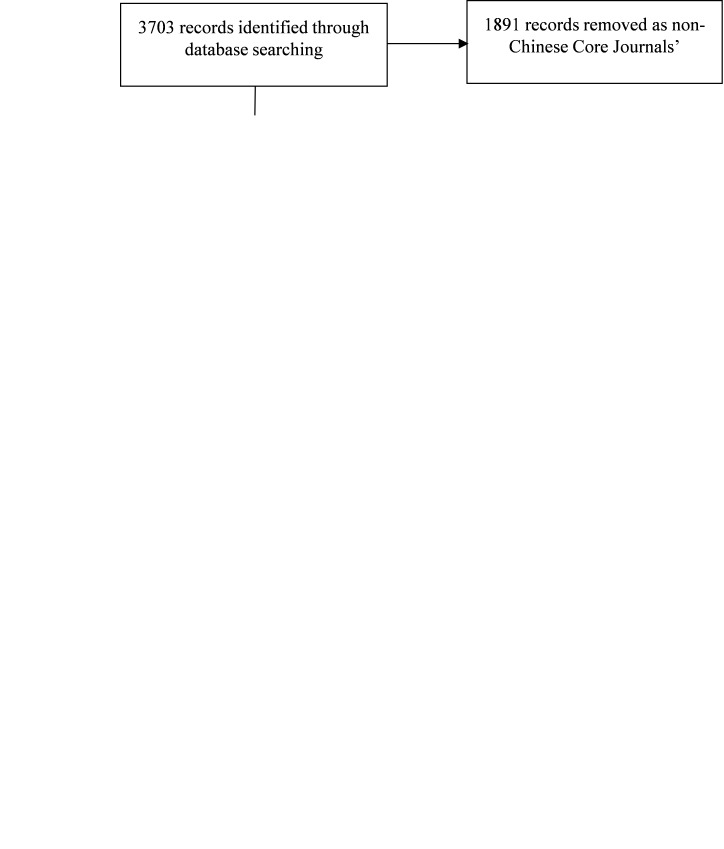
Flow diagram of literature search and selection.

The electronic search identified 3703 citations (**Figure 1**). After removal of non–Chinese core journals and duplicates, and screening titles and abstracts, 90 studies were judged as potentially relevant with the full text retrieved to assess their eligibility for inclusion. Overall, 50 studies [17–66] met the eligibility criteria and were thus included in our analysis. These studies reported data collected from 1991 to 2013. The quality evaluation score for these studies ranged from 5 to 10 points, with a mean ± standard deviation of 8.1 ± 1.2. There were 36 (72%) studies with a score of ≥8. A summary of the characteristics of included studies associated with their respective quality assessment is presented in Appendix S2 in **Online Supplementary Document(Online Supplementary Document)**. Studies included the analysis are listed in Appendix S3 in **Online Supplementary Document(Online Supplementary Document)**.

### Meningococcal disease incidence and associated mortality rate

Eleven studies [21,24,25,29,44,46,48,57–60] conducted in 7 provinces provided data on the meningococcal disease incidence and associated mortality rate. The annual meningococcal disease incidence and mortality rate for a representative period between 2000 and 2010 is summarized in **Table1**. The highest incidence rates occurred in the years 2006, 2007 and 2010; the incidence rates reported in these years were >2.0 per 100 000 persons per year. The corresponding highest mortality rates occurred in the years 2002, 2003 and 2010; the mortality rate reported in these years was 0.44 per 100 000 persons per year. The average incidence of meningococcal disease and associated mortality during the study period assessed were estimated to be 1.84 (95% confidence interval (CI) 0.91–3.37) per 100 000 and 0.33 (95% CI 0.12–0.86) per 100 000, respectively.

**Table 1:** Meta–analysis of annual meningococcal disease incidence and associated mortality rate from 2000 to 2010 (per 100 000 persons per year)

Year	Incidence		Mortality	
**Number of studies**	**Rate**	**Number of studies**	**Rate**
2000	5	0.71 (0.18–2.44)	3	0.37 (0.02–3.79)
2001	5	0.66 (0.17–2.28)	3	0.01 (0.00–9.96)
2002	5	0.91 (0.23–3.00)	3	0.44 (0.06–2.52)
2003	6	1.23 (0.31–3.85)	3	0.44 (0.66–2.52)
2004	7	1.09 (0.19–4.33)	3	0.02 (0.00–9.18)
2005	10	1.07 (0.26–3.53)	4	0.02 (0.00–7.82)
2006	8	2.18 (0.69–5.13)	4	0.02 (0.00–7.68)
2007	8	2.30 (0.76–5.21)	4	0.01 (0.00–8.91)
2008	5	1.41 (0.17–6.14)	3	0.01 (0.00–9.97)
2009	5	1.88 (0.53–4.89)	3	0.40 (0.03–4.01)
2010	3	2.17 (0.06–5.42)	2	0.44 (0.03–4.35)
Total	11	1.84 (0.91–3.37)	5	0.33 (0.12–0.86)

### *N. meningitidis* carriage rate among the healthy population

Twenty–nine studies [17–19,22,24,26–28,30–32,34,35,37,39–44,47,49,51,52,56,57,62,64,65] conducted in 14 provinces reported *N. meningitidis* carriage rates among the healthy population. The analysis for *N. meningitidis* carriage rate included 1248 positive cases identified from 45 462 throat swabs from healthy people between 2000 and 2013, representing an overall carriage rate of 2.7% (95% CI 2.0%–3.5%).

### Prevalence of antibodies against *N. meningitidis* among the healthy population

Twenty–three studies [20,22,23,28,30,33,34,36,38,39,42,43,45,50,52–55,57,61,63,65,66] conducted in 11 provinces reported the prevalence of antibodies against *N. meningitidis* among the healthy population. The age–specific prevalence of antibodies *against N. meningitidis* is summarized in **Table 2**. Serogroup A specific *N. meningitidis* were generally highest in those aged 5–24 years and for serogroup C it was highest in those aged 25 years or older. The overall prevalence of antibodies against *N. meningitidis* serogroup A and C was 77.3% (95% CI 72.4%–81.6%) and 33.5% (95% CI 27.0%–40.8%), respectively, for the period 2001–2012.

**Table 2:** Meta–analysis of age–specific prevalence of antibodies against *N. meningitidis* among the healthy population

Age–group (years)	Number of studies	Antibody–positive	Total number of participants	Positive rate (%) (95% CI)
**Meningococcal serogroup A:**				
0–4	22	3101	4701	70.9 (63.4–77.4)
5–9	15	1367	1722	81.4 (75.5–86.2)
10–14	15	1515	1912	78.2 (72.4–83.1)
15–24	15	1623	2071	78.2 (71.7–83.6)
25–34	14	1101	1488	75.0 (66.1–82.2)
35–44	14	1058	1357	76.3 (65.3–84.7)
45–	14	1038	1424	72.5 (61.6–91.2)
Total	22	10 803	14676	77.3 (72.4–81.6)
**Meningococcal serogroup C:**				
0–4	22	1658	4395	23.6 (16.0–33.5)
5–9	14	610	1367	34.9 (23.5–48.4)
10–14	14	533	1347	24.1 (14.7–36.9)
15–24	14	606	1539	32.0 (24.4–40.7)
25–34	13	616	1347	39.9 (31.9–48.5)
35–44	13	656	1210	42.6 (32.2–53.7)
45–	13	703	1283	46.7 (37.0–56.6)
Total	22	5382	12 488	33.5 (27.0–40.8)

CI – confidence interval

### Sensitivity analysis and publication bias

Sensitivity analyses undertaken to include only ‘good’ quality studies did not significantly alter the outcomes. Overall *N. meningitidis* carriage rate among the healthy population reported in 22 good quality studies was 3.2% (95% CI, 2.4%–4.2%) and the prevalence of antibodies against *N. meningitidis* serogroup A and C among the healthy population reported in 20 good quality studies were 75.9% (95%CI 70.5%–80.6%) and 34.3% (27.5%–42.0%), respectively. The Egger’s test did not reveal any significant publication bias (–0.51, 95% CI –4.72 to 2.87, *P* = 0.616) for *N*. *meningitidis* carriage rate among the healthy population. As the majority of studies were captured in the analysis of carriage data, we did not consider further sensitivity analysis of the other data to be necessary.

### Discussion

Our study is the first to systematically review, collate and analyze available published studies on the disease burden of *N. meningitidis* infections in China using robust meta–analytical methods. We found that the incidence of meningococcal disease and associated mortality are low ranging 0.66–2.30 per 100 000 persons per year and 0.01–0.44 per 100 000, respectively. The incidence of meningococcal disease from our study is consistent with that reported in developed countries, typically <2 per 100 000, but lower than that reported in developing countries (typically >10 per 100 000), particularly in Africa [67].

In China, meningococcal disease historically occurred in a cyclical pattern at intervals of 8–10 years, with nationwide epidemics in 1959, 1967, 1977, and 1984 [7,68]. The spring of 1967 had the highest incidence of meningococcal disease with reported rates of 403 per 100 000, corresponding to more than 3.04 million cases. The associated mortality rate in that year was 5.5% corresponding to more than 160 000 deaths [69]. The epidemic in 1977 had an incidence of 59.7 per 100 000 and a 4.0% fatality rate. These deadly cyclic epidemics and seasonal patterns clearly highlight the unpredictability of outbreaks of meningococcal disease despite our observed low incidence rates in the study period.

In our analysis, the prevalence of antibodies against *N. meningitidis* serogroup C was 33.5% and tended to increase with age, whereas the prevalence of serogroup A antibodies was highest in the 5–9 year age group and decreased with age. The lower seroprevalence of serogroup C within the population suggests that it may pose a greater risk for meningococcal disease outbreak than serogroup A, particularly in the youngest age group (less than 5 years) who have the lowest seroprevalence. Despite over 90% uptake for meningococcal serogroup A/C polysaccharide vaccines, the observation of low seroprevalence against serogroup A and C in children less than 5 years old suggests that the implementation of conjugate meningococcal vaccine is necessary particularly for those less than 2 years old to whom the polysaccharide meningococcal vaccines have limited benefits and protection.

We estimated the nasopharyngeal *N. meningitidis* carriage to be 2.7%, which appear lower than the generally quoted overall rate of 10% [70], and the average 3.5–35% reported in studies conducted in Africa [71], but at least consistent with that reported in Mexico (1.6%) [72]. The low carriage rates found in our study and those reported in Mexico are consistent with the low disease incidence rate reported in these two countries. Currently the burden of meningococcal disease in Mexico is low, with total national cases as low as two per year [73,74]. The prevalence of *N. meningitidis* carriage in healthy children and adolescents aged 10–19 years in Chile was reported to be 6.5% [75], and slightly lower in university students ages 18–24 years (4%) [76]. The corresponding incidence of meningococcal disease in Chile was also low, varying from 0.33–0.59 per 100 000 in the six years up to 2012 [77]. A European meta–analysis including 143 114 individuals found that the carriage rate increased from 4.5% in infants to 24% in 19–year olds and decreased to 8% in 50–year old adults [78]. Data on age–specific carriage rates in China are currently lacking. Since information on carriage rates is important for understanding the epidemiology and transmission of meningococcus and developing vaccination strategies, studies on age–specific carriage rates are recommended in China.

Our study has a number of limitations that should be considered. The meta–analysis was based on observational studies and as such is constrained by the inherent heterogeneity in such studies (for example, differences in sampling techniques, laboratory methods, age groups assessed, period of study/seasonality effects) and underlying confounding factors. In addition, we combined data from different regions and time periods. To account for this, we adopted a random–effects model to pool all results, leading to a wider 95% CI that provided a more conservative estimate of the overall results. There were few studies found for certain geographic regions of west China, such as Gansu, Xinjiang and Tibet, which may also contribute to low precision estimates in those areas and overall. We did not search the gray literature; therefore, data that were not published in the 4 selected search databases may have been missed. Nevertheless, our study is likely to capture all important Chinese data on meningococcal disease since we included all available core journals in our study. Other shortcoming are, we only considered studies published in English and Chinese and did not contacted to authors for missing information or resolve ambiguities. However, it is unlikely there would be any significant literature in other languages. Since most important study data were available to obtain during data extraction, we feel that clarifications with the authors are not necessary and do not expect to have any impact on the study results. In addition, there were no studies identified that reported disease burden by serogroup, which limits our understanding of seroepidemiology of meningococcal disease and for developing recommendations for the selection of meningococcal vaccines in different age groups. Nevertheless, almost all meningococcal disease was caused by serogroup A and C based on the limited available data [11,79]. Therefore, the use of meningococcal conjugate vaccines including serogroups A/C/W/Y can further reduce the burden of meningococcal disease and prevent the occurrence of large outbreaks in China.

In conclusion, although the overall incidence of meningococcal disease in China is low, the lower seroprevalence of serogroup C within the population suggest that it may pose a greater risk for meningococcal disease outbreak than serogroup A, especially for children aged less than 5 years. The lack of data on serogroup–specific disease burden by age group suggests that the implementation of laboratory–based meningococcal surveillance systems is urgently needed in China.
