# Supplementary material for: Burden of Neisseria meningitidis infections in China: a systematic review and meta–analysis
Source: J Glob Health. 2016 Nov 7;6(2):020409. doi: 10.7189/jogh.06.020409 (PMC5112005; doi:10.7189/jogh.06.020409)
Supplement: Online Supplementary Document [file jogh-06-020409-s001.pdf]

Online Supplementary Document

Kyaw et al. Burden of Neisseria meningitidis infections in China: a systematic review and meta–analysis

J Glob Health 2016;6:020409

Appendix S1. Search strategy and results

Total literatures: 3713 Aug 5, 2015

1）CNKI：1091篇

SU=(‘脑膜炎双球菌’+‘脑膜炎球菌’+‘脑膜炎奈瑟菌’+‘脑膜炎奈瑟氏菌’+‘脑膜炎奈瑟氏球菌’+‘脑膜炎奈瑟球菌’+‘脑脊髓膜炎双球菌’+‘脑脊髓膜炎球菌’+‘脑脊髓膜炎奈瑟菌’+‘脑脊髓膜炎奈瑟氏菌’+‘脑脊髓膜炎奈瑟氏球菌’+‘脑脊髓膜炎奈瑟球菌’+‘meningococcus’+‘meningococcal’+‘meningococcic’+‘meningitides’)

学科领域：医药卫生科技
检索日期：2005/01/01-2015/08/05

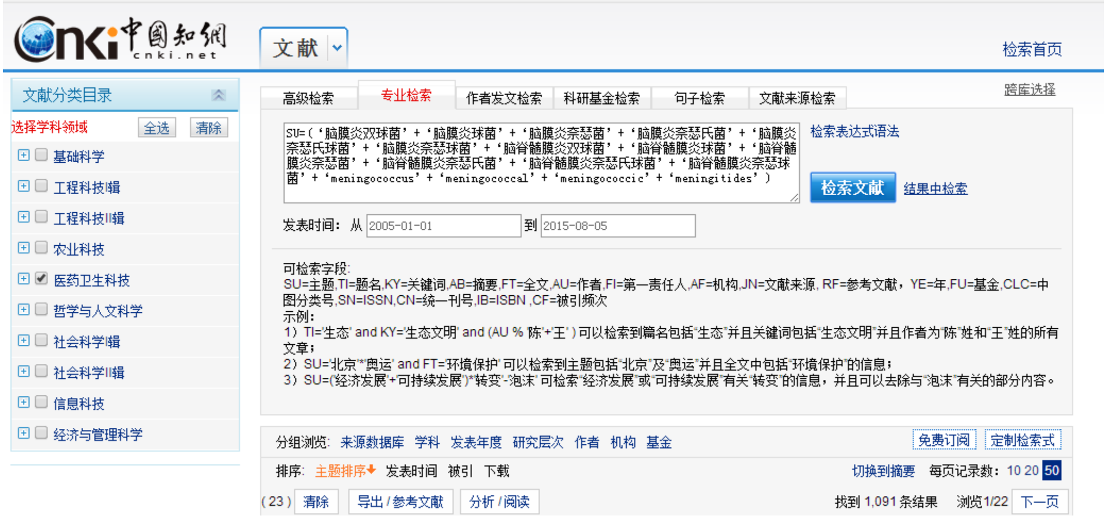

2）万方：985篇

主题:("脑膜炎球菌") + 主题:("脑膜炎双球菌") + 主题:("脑膜炎奈瑟菌") + 主题:("脑膜炎奈瑟氏菌") + 主题:("脑膜炎奈瑟氏球菌") + 主题:("脑膜炎奈瑟球菌")+ 主题:("脑脊髓膜炎球菌") + 主题:("脑脊髓膜炎双球菌") + 主题:("脑脊髓膜炎奈瑟菌") + 主题:("脑脊髓膜炎奈瑟氏菌") + 主题:("脑脊髓膜炎奈瑟氏球菌") + 主题:("脑脊髓膜炎奈瑟球菌") + 主题:("meningococcus") +主题:("meningococcal") + 主题:("meningococcic") +主题:("meningitides")

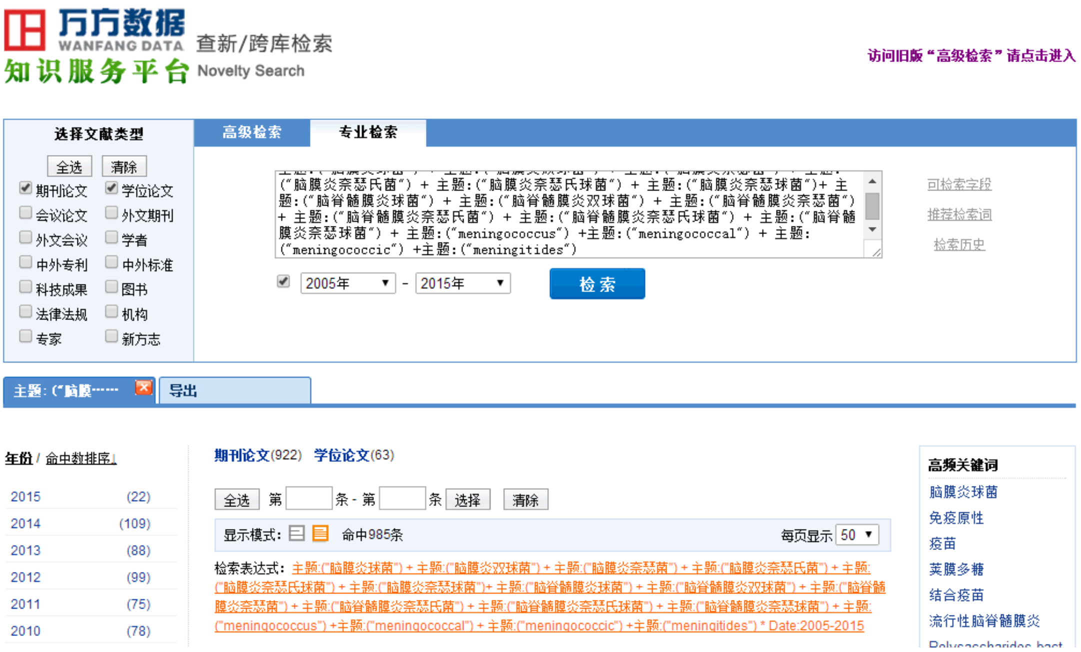

3）中国生物医学文献（CBM）：1577篇

((((((((((((((("脑膜炎球菌"[常用字段] OR ("脑膜炎双球菌"[常用字段] OR "脑膜炎奈瑟球菌"[常用字段] OR "奈瑟球菌, 脑膜炎"[主题词])) OR "脑膜炎奈瑟菌"[常用字段]) OR "脑膜炎奈瑟氏菌"[常用字段]) OR "脑膜炎奈瑟氏球菌"[常用字段]) OR "脑膜炎奈瑟球菌"[常用字段]) OR "脑脊髓膜炎球菌"[常用字段]) OR "脑脊髓膜炎双球菌"[常用字段]) OR "脑脊髓膜炎奈瑟菌"[常用字段]) OR "脑脊髓膜炎奈瑟氏菌"[常用字段]) OR "脑脊髓膜炎奈瑟氏球菌"[常用字段]) OR "脑脊髓膜炎奈瑟球菌"[常用字段]) OR ("meningococcus"[常用字段] OR "脑膜炎奈瑟球菌"[常用字段] OR "脑膜炎双球菌"[常用字段] OR "奈瑟球菌, 脑膜炎"[主题词])) OR "meningococcal"[常用字段]) OR "meningococcic"[常用字段])) AND 2005-2015[日期]

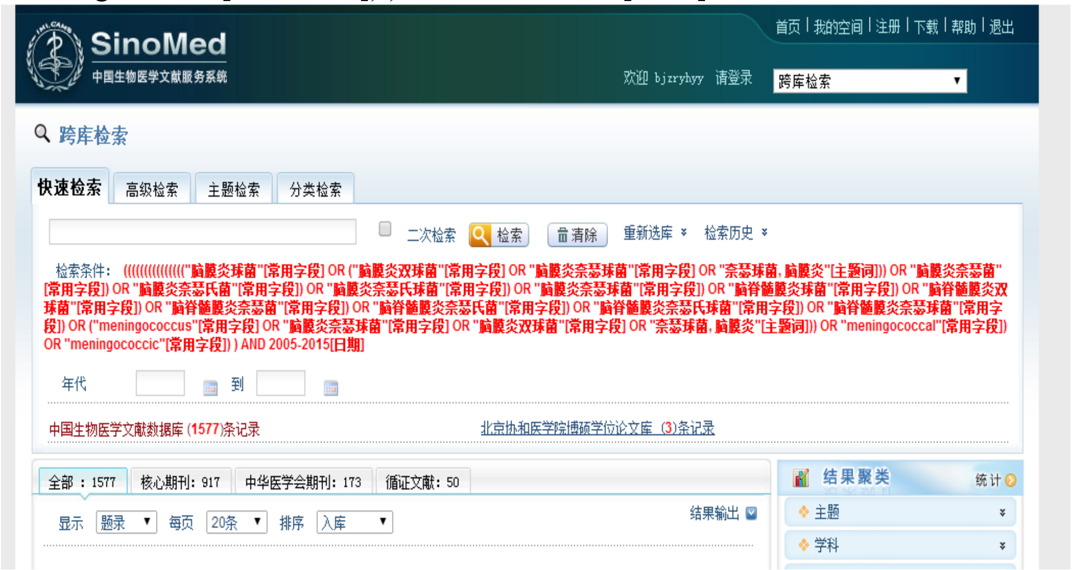

4）PubMed：60篇

(meningococcus[Title/Abstract] OR meningococcal[Title/Abstract] OR meningococcic[Title/Abstract] OR meningitides[Title/Abstract] OR meningitidis[Title/Abstract]) AND (Chinese[Title/Abstract] OR China[Title/Abstract]) AND

(("2005/01/01"[PDAT] : "2015/08/05"[PDAT]) AND "humans"[MeSH Terms])

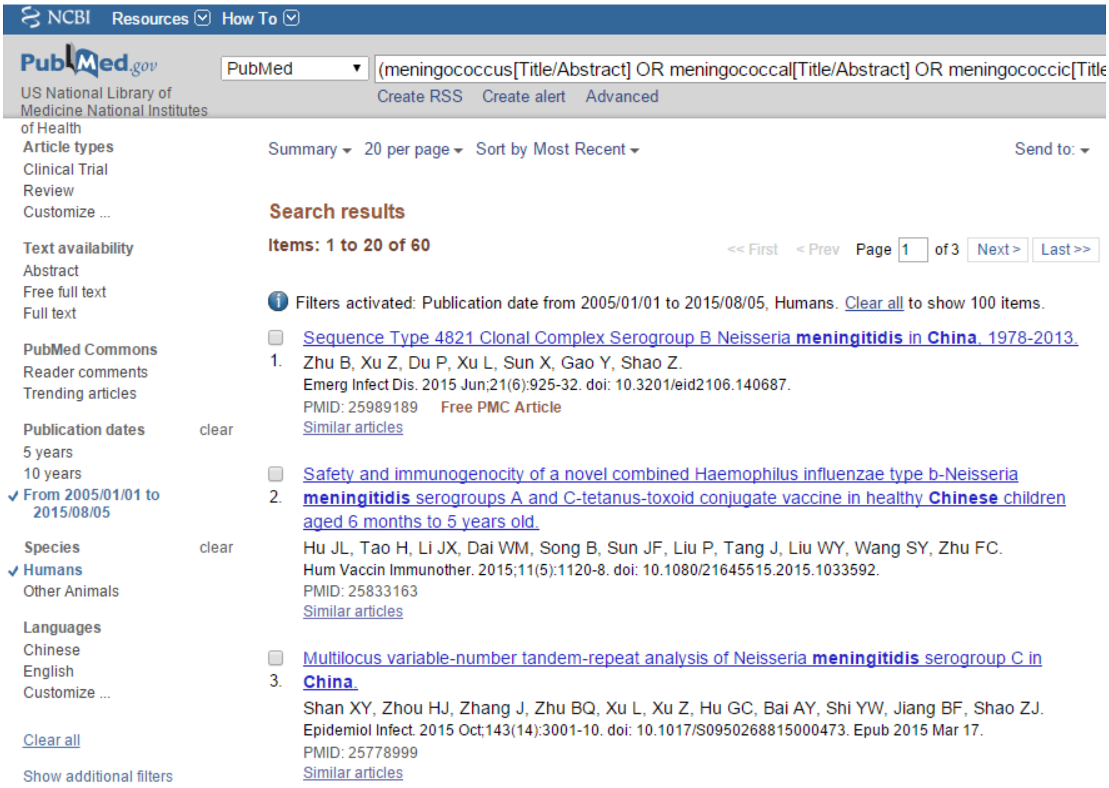

NCBI Resources How To

PubMed.gov
US National Library of Medicine National Institutes of Health

PubMed (meningococcus[Title/Abstract] OR meningococcal[Title/Abstract] OR meningococcic[Title

Create RSS Create alert Advanced

Article types
Clinical Trial
Review
Customize ...

Text availability
Abstract
Free full text
Full text

PubMed Commons
Reader comments
Trending articles

Publication dates clear
5 years
10 years
✓ From 2005/01/01 to 2015/08/05

Species clear
✓ Humans
Other Animals

Languages
Chinese
English
Customize ...

Clear all

Show additional filters

Summary 20 per page Sort by Most Recent Send to:

Search results

Items: 1 to 20 of 60

<< First < Prev Page 1 of 3 Next > Last >>

Filters activated: Publication date from 2005/01/01 to 2015/08/05, Humans. Clear all to show 100 items.

1. Sequence Type 4821 Clonal Complex Serogroup B Neisseria **meningitidis** in **China**, 1978-2013.
Zhu B, Xu Z, Du P, Xu L, Sun X, Gao Y, Shao Z.
Emerg Infect Dis. 2015 Jun;21(6):925-32. doi: 10.3201/eid2106.140687.
PMID: 25989189 Free PMC Article
Similar articles

2. Safety and immunogenocity of a novel combined Haemophilus influenzae type b-Neisseria **meningitidis** serogroups A and C-tetanus-toxoid conjugate vaccine in healthy **Chinese** children aged 6 months to 5 years old.
Hu JL, Tao H, Li JX, Dai WM, Song B, Sun JF, Liu P, Tang J, Liu WY, Wang SY, Zhu FC.
Hum Vaccin Immunother. 2015;11(5):1120-8. doi: 10.1080/21645515.2015.1033592.
PMID: 25833163
Similar articles

3. Multilocus variable-number tandem-repeat analysis of Neisseria **meningitidis** serogroup C in **China**.
Shan XY, Zhou HJ, Zhang J, Zhu BQ, Xu L, Xu Z, Hu GC, Bai AY, Shi YW, Jiang BF, Shao ZJ.
Epidemiol Infect. 2015 Oct;143(14):3001-10. doi: 10.1017/S0950268815000473. Epub 2015 Mar 17.
PMID: 25778999
Similar articles

Appendix S2. Summary of Articles used for Meta-analysis

Author	Study Period	Province	Study Design	No. of cases	Incidence and Mortality rate	Carriage rate	Antibody level	Quality Assessment*					
								Q1	Q2	Q3	Q4	Q5	Total
Zhou ZR[17]	2008	Sichuan	Cross-sectional	887		YES		2	2	2	2	1	9
Zhang XC[18]	Not Specified	Sichuan	Cross-sectional	999		YES		2	2	2	2	0	8
Li Q[19]	2008	Sichuan	Cross-sectional	321		YES		2	1	1	2	0	6
Zhang XQ[20]	2005	Guangdong	Surveillance	205			YES	2	2	1	2	0	7
Dai B[21]	2005-2007	Anhui	Surveillance	-	YES			1	1	2	2	1	7
Zheng CZ[22]	2007	Jiangsu	Cross-sectional	442		YES	YES	2	2	1	2	1	8
Zheng CZ[23]	2007	Jiangsu	Cross-sectional	897			YES	2	2	1	2	1	8
Feng L[24]	2006-2013	Shandong	Surveillance	13712	YES	YES		2	2	1	2	2	9
Zhang TG[25]	2005	Beijing	Surveillance	-	YES			1	2	2	2	1	8
Zhou ZR[26]	2009	Sichuan	Cross-sectional	107		YES		2	1	2	2	1	8
Long DL[27]	2008-2009	Sichuan	Cross-sectional	614		YES		2	1	2	2	1	8
Lin Z[28]	Not Specified	Neimenggu	Cross-sectional	181		YES	YES	2	2	1	2	1	8
Long J[29]	2000-2005	Chongqing	Surveillance	-	YES			1	1	1	2	1	6
Luo XC[30]	2005	Fujian	Cross-sectional	360		YES	YES	2	2	2	2	1	9
Luo SH[31]	2004-2005	Guangdong	Cross-sectional	1355		YES		1	1	2	2	1	7
Luan L[32]	2005-2012	Jiangsu	Surveillance	4043		YES		2	1	1	2	1	7
Jiang X[33]	2008	Hebei	Cross-sectional	702			YES	2	1	2	2	2	9
Liu MZ[34]	2005	Guangdong	Cross-sectional	1077		YES	YES	2	2	2	2	1	9

Tang XL[35]	2007	Qinghai	Cross-sectional	480		YES		2	2	2	2	1	9
Yang HK[36]	2005	Guangdong	Surveillance	175			YES	1	1	2	2	2	8
Yang Y[37]	2007	Sichuan	Cross-sectional	230		YES		2	2	2	2	2	10
Jiang WP[38]	2004	Jiangsu	Cross-sectional	855			YES	2	1	2	2	2	9
Wang J[39]	2006	Ningxia	Cross-sectional	214		YES	YES	2	1	2	2	1	8
Fan YS[40]	2001-2007	Hebei	Cross-sectional	1792		YES		1	2	1	2	1	7
Wu XH[41]	2008	Guangxi	Cross-sectional	367		YES		2	2	2	2	2	10
Liu DQ[42]	2004-2006	Anhui	Surveillance	2246		YES	YES	2	2	1	2	2	9
Ju CY[43]	2005-2006	Guangdong	Cross-sectional	1255		YES	YES	2	1	2	2	2	9
Ni JD[44]	2000-2007	Anhui	Surveillance	2493	YES	YES		2	2	1	1	2	8
Xu BX[45]	2004-2005	Zhejiang	Cross-sectional	4584			YES	2	1	2	2	2	9
Xu XH[46]	2000-2010	Anhui	Surveillance	-	YES			2	2	1	2	2	9
Zhang JM[47]	2003-2008	Zhejiang	Cross-sectional	1507		YES		2	2	2	2	1	9
Dai B[48]	2003-2007	Anhui	Surveillance	-	YES			2	2	1	2	2	9
Wang YT[49]	2006-2012	Hebei	Cross-sectional	577		YES		2	2	2	2	2	10
Jiang X[50]	2006-2008	Hebei	Cross-sectional	1934			YES	2	2	2	2	2	10
Lv J[51]	2006-2010	Hubei	Cross-sectional	2908		YES		1	2	1	2	2	8
Li QW[52]	2012	Fujian	Cross-sectional	806		YES	YES	2	2	1	2	1	8
Li YC[53]	2010	Guangdong	Cross-sectional	249			YES	2	2	2	2	2	10

Li WD[54]	2009	Guangdong	Cross-sectional	294			YES	2	2	2	2	2	10
Chen W[55]	2007	Tianjin	Cross-sectional	1062			YES	2	2	1	2	2	9
Hou TJ[56]	2011-2012	Shanxi	Surveillance	411		YES		2	1	1	2	1	7
Zhang H[57]	2004-2005	Jiangsu	Surveillance	468	YES	YES	YES	2	1	1	1	2	7
Fan YC[58]	1991-2010	Neimenggu	Surveillance	-	YES			2	1	0	0	2	5
Jiang HB[59]	2009	Jiangsu	Surveillance	-	YES			2	2	0	0	2	6
Ma HS[60]	2005-2009	Hebei	Surveillance	-	YES			2	2	0	1	2	7
Mo SX[61]	2007	Hainan	Surveillance	614			YES	2	2	1	1	2	8
Yin HJ[62]	2005	Beijing	Cross-sectional	1010		YES		2	0	1	1	2	6
Gu WW[63]	2006	Hubei	Cross-sectional	424			YES	2	2	1	1	1	7
Wang XP[64]	2004-2006	Anhui	Surveillance	2821		YES		2	2	1	1	2	8
Guo F[65]	2001-2006	Hubei	Surveillance	1812		YES	YES	2	2	1	2	1	8
Liao GD[66]	2005	Guangdong	Cross-sectional	195			YES	2	2	2	1	2	9

*Q1. Did the publication report participants' source exactly?
Q2. Did the publication report diagnosis criteria completely?
Q3. Did the publication report specimen collection methods?
Q4. Did the publication report pathogen or antibody detection methods?
Q5. Did the publication report statistical methods?

Appendix 3.

S1. Zhou ZR, Liang JL, Long DL, Li Q, Zhao XZ, Liu HC. Carrying Rate of Neissria Meningitides in a healthy population in the Mianzhu Post-earthquake residential area. J Sichuan Univ (Med Sci Edi) 2009;40:716-8.

S2. Zhang XC, Zhu KR, Huang RN, Long HY. Evaluation of a rapid real-time PCR assay for detection the bacteria carrier level of Neisseria Meningitides in health population in Chengdu city. Modern Preventive Medicine 2009;36:1710-2.

S3. Li Q, Wang XL Zhang YM, Yang H, Zhao XZ. A survey of taking Neissria Meningitides in victims of Wenchuan earthquake in Mianzhu. Chinese Journal of Health Laboratory Technology 2008;18:2723-4.

S4. Zhang XQ, Deng ZA, Zhou XZ, Li XQ, Li CH, Lin YW. Investigation on the immune level of the Menitis bacterium types A and C among the normal people in Fanyu, Guangzhou. Chinese Journal of Health Laboratory Technology 2005;15:1360-1.

S5. Dai B, Ni JD, Jin YH, Bai RJ, Zheng N, Ye DQ. Epidemiological characteristics of Meningococcal Meningitis in Hefei city during 2005 and 2007. Chin J Public Health 2009;25:79-80.

S6. Zheng CZ, Shao RB, Bi C, Xun YB, Ma YF. Surveillance for the carrier of Neisseria Meningitis group A and C and antibody level among healthy people in Yancheng city. Modern Preventive Medicine 2012;39:1541-2,1552.

S7. Zheng CZ, Wang B, Shao RB, Bi C, Chen CB, Cai JP, et al. Investigation on the immune level of the Menitis bacterium types A and C among healthy people. Modern Preventive Medicine 2009;36:3949-50,3952.

S8. Feng L, Xiong P, Li MS, Song LZ, Zhang Y, Liu GF, et al. Analysis on the epidemiological characteristics and the etiological surveillance for epidemic cerebrospinal Meningitis in Shandong province from 2006 to 2013. Chin J Epidemiol 2014;35:1407-8.

S9. Zhang TG, He X, Chen LJ, He JG, Yang J, Sun MP, et al. Surveillance on pathogens of Meningococcal Meningitis in Beijing, 2005. Chin J Epidemiol 2006;27:396-8.

S10. Zhou ZR, Zhao XZ, Long DL, Deng Q, Li Q, Liu HC. Survey of germ-carrying rate of Neissria Meningitides in one prefab community in disaster Area. Modern Preventive Medicine 2010;37:3338-40.

S11. Long DL, Yao P, Gao WF, Wang ZX, Gao RR, Zhou ZR, et al. Survey on prevalence of Neisseria Meningitides among students in primary and middle schools in SiChuan distress area. Modern Preventive Medicine 2011;38:476-7,481.

S12. Lin Z, Liu YY, Ding C, Chang XL, Liu TY, Li JC, et al. Survey of Neissria mealingitidis Carrier in healthy people in Tongliao. Chin J Public Health 2006;22:1500.

S13. Long J, Zhao H, Li Q, Luo XJ, Xiao BZ, Yi J. Analysis of surveillance on epidemic cerebrospinal Meningitis from 2000 to 2005 in ChongQing city. Modern Preventive Medicine 2007;34:1652-5.

S14. Luo XC, Luo LM, Gong Y, Luo HH, Cai SW, Zhao Q. The prevalence characteristics of epidemic cerebrospinal Meningitis in Sanming city in 2005. Chinese Journal of Health Laboratory Technology 2006;16:451-2.

S15. Luo SH, Zhang LP, Huang SZ, Li JQ. The prevalence characteristics of epidemic cerebrospinal Meningitis from 2000 to 2005 in Dongguan city. Chinese Journal of Health Laboratory Technology 2006;16:701-2.

S16. Luan L, Zhan YH, Zhang MH, Bi C, Zhu YH, Zhang J, et al. Epidemiological analysis of the Meningococcal Disease in Suzhou city during 1992-2012. Modern Preventive Medicine 2014;41:2113-5,2118.

S17. Jiang X, Qian ZY, Zhen SU, Wang YT, Jia ZY, Sun YQ, et al. Analysis of the antibody level of epidemic cerebrospinal Meningitis among healthy population in HeBei province in 2008. Modern Preventive Medicine 2011;38:3415-7.

S18. Liu MZ, Yang HK, Liao GD, Huang ZS, Xu L, Deng XL, et al. Investigation on antibody level against epidemic cerebrospinal meningitis and carriage rate of Neisseria Meningitidis in healthy population in Guangdong province. Chinese Journal of Health Laboratory Technology 2007;17:325-6,338.

S19. Tang XL. Investigation on germ-carrying situation on Neisseria Meningitidis of healthy populations in Qinghai province in 2007. Modern Preventive Medicine 2010;37:555-6.

S20. Yang HK, Zhang LP, Liu MZ. Surveillance analyses on epidemic cerebrospinal Meninqitis of Dongguan in 2005. Chinese Journal of Health Laboratory Technology 2006;16:76-7.

S21. Yang Y, Liang JL, Ju CY, Su HW, Chen ZQ, Gan ZL, et al. Investigation on the germ-carrying status of epidemic Meningitis among health rural students in primary and middle school of Luzhou. Modern Preventive Medicine 2008;35:2347-9.

S22. Jiang WP, Wang YJ. Analysis on antibody level against epidemic cerebrospinal Meningitis serogroup A

and C in Danyang. Chin J Dis Control Prev 2005;9:348-9,377.
S23. Wang J, Yan LQ. Surveillance analyses on epidemic cerebrospinal Meninqitis of Zhongwei city of Ningxia in 2006. Chinese Journal of Health Laboratory Technology 2007;17:495-6.
S24. Fan YS, Zhang LS, Wang JH. Survey of N. mealingitidis Carrier in healthy people in Tangshan. Chinese Journal of Health Laboratory Technology 2008;18:690-1.
S25. Wu XH, Lin J, Quan Y, Fang JS, Liang DB, Qin WW, et al. Research on bacteria carrying, antibody level and control strategy for epidemic cerebrospinal Meningitis in Guangxi. Chin J Sch Health 2010;31:337-9.
S26. Liu DQ, Wang JJ, Wang BB, Chen X, Luo XW, Yan KL, et al. The prevalence characteristics of epidemic cerebrospinal Meningitis of serogroup C in Anhui Province. Chin J Dis Control Prev 2010;14:240-4.
S27. Ju CY. Genetic relationships among carried meningococci and the immunity of healthy people in Shenzhen, China. Sichuan University. 2007.
S28. Ni JD. Study on the epidemiological characteristics of Meningococcal disease in Anhui province. Anhui Medical University. 2008.
S29. Xu BX, Yao PP, Wang FS. Analysis and survey of antibodies against neisseria meningitides in healthy population in Zhejiang province. Chinese Journal of Health Laboratory Technology 2013;23:195-9.
S30. Xu XH. Epidemiology and molecular epidemiology of Meningococcal Meningitis during 2000-2010 in Hefei city of Anhui province. Anhui Medical University. 2014.
S31. Zhang JM, Jin WE, Ye CH. Situation of carrier rate of Neisseria Meningtitides in school health population in Quzhou city during 2003-2008. Chinese Journal of Health Laboratory Technology 2013;19:1647-8,1669.
S32. Dai B. Study on the epidemiological characteristics of Meningococcal disease in Hefei city. Anhui Medical University. 2009.
S33. Wang YT. Survey on carriage of Neisseria Meningitidis, Haemophilus Influenzaes, Streptococcus Pneumoniae among healthy population in Hebei province. Chinese Center fro Disease Control and Prevention. 2013.
S34. Jiang X, Sun YQ, Qian ZY, Zhen SJ, Wang YT, Jia ZY, et al. Surveillance on antibody level against Meningcoccus among healthy population in three city of Hebei province. Chinese Journal of Health Laboratory Technology 2011;21:164-6.
S35. Lv J, Yang HM, Jiang YZ, Zou WJ, Zhao MJ, Zhu BQ, et al. Analysis of the result on surveillance of epidemic cerebrospinal Meningitis in Hubei, 2006-2010. Journal of Pathogen Biology 2012;7:287-90.
S36. Li QW, Yuan WH, Zheng EH, Yuan L, Lin ZY, Chen AP. Monitoring and analysis of epidemic cerebrospinal Meningitis infection rate and antibody level in healthy population in Fujian province in 2012. Chinese Journal of Health Laboratory Technology 2014;24:244-6.
S37. Li YC, Huang SZ, Ye XY, Zhang LP, Yang L, Fang CY. Analysis on serum bactericidal antibody level of Neisseria Meningtides serogroup C in healthy population of Dongguan city in 2010. Chinese Journal of Health Laboratory Technology 2012;22:2957-8,2965.
S38. Li WD, Li JM, Yang KX. Analysis on surveillance of antibody level of groups A and C epidemic cerebrospinal Meningitis of healthy population in Longgang of Shenzhen. Modern Preventive Medicine 2010; 37:2103-4,2108.
S39. Chen W, Jin LY, Qi XY. Investigation on antibody levels against epidemic cerebrospinal Meningitis and appraisal on immune efficacy among the healthy population in Tianjin. Modern Preventive Medicine 2010; 37:3738-9,3749.
S40. Hou TJ, Wang LL. Epidemiological characteristics and disease control of meningitis in Xi'an. Chin J Microbiol Immunol 2014;34:713-5.
S41. Zhang H, Hang H, Xu QY. Epidemiological characteristics of cerebrospinal Meningitis in Suzhou. Modern Preventive Medicine 2006; 33:1864-6.
S42. Fan YC, Li C, Yan Z, Yan SH. Surveillance and analysis of epidemic cerebrospinal Meningitis in Inner Mongolia autonomous region during 1991-2010. Modern Preventive Medicine 2012; 39:5527-8,5531.
S43. Jiang HB. Analysis of monitoring result of epidemic cerebrospinal Meningitis in Lianyungang City during 2004-2009. Modern Preventive Medicine 2012; 39:1078-80.
S44. Ma HS, Sun YQ, Liu HB, Li YP, Zhen SJ, Jiang X, et al. Analysis of epidemic cerebrospinal Meningitis deaths in Hebei province during 2005-2009. Modern Preventive Medicine 2011; 38:833-4,839.
S45. Mo SX, Sun LY, Zeng XX, Fu ZW, He J, Li J, et al. Analysis on the cerebrospinal Meningitis antibody level of health population in Hainan in 2007. Modern Preventive Medicine 2010; 37:2913-5.

S46. Yin HJ, Shi J, Gao GH, Xu XM, Chu G, Qiu J, et al. Analysis of knowledge and health situation of epidemic cerebrospinal meningitis carrier among passengers. Chin J Prev Med 2008;42:139-41.
S47. Gu WW. Investigation on the immune level of the Meningitis types A and C among healthy people in Shiyan city. Modern Preventive Medicine 2008; 35:3193,3196.
S48. Wang XP, Jin YH, Wang H. Analysis on epidemic characteristics of epidemic cerebrospinal meningitis from 2004- 2006 in Hefei. Modern Preventive Medicine 2008; 35:2011-3.
S49. Guo F, Liu XH, Jiang B, Peng B, Huang XM, Wang YG, et al. Analysis on the surveillance results of epidemic cerebrospinal meningitis in the healthy people in Suizhou city from 2001 to 2006. Chin J Dis Control Prev 2007;11:482-4.
S50. Liao GD, Liao XJ, Liu MZ, Luo XM. Investigation on the immune level of the Menitis bacterium types A and C among healthy people in Maoming, Guangdong. Chin J Epidemiol 2006;27:180.
